# CRISPR disruption of TCTP gene impaired normal development in the silkworm *Bombyx mori*

**DOI:** 10.1111/1744-7917.12567

**Published:** 2018-02-19

**Authors:** Zu‐Lian Liu, Jun Xu, Lin Ling, Ru Zhang, Peng Shang, Yong‐Ping Huang

**Affiliations:** ^1^ Faculty of Life Sciences Northwestern Polytechnical University Xi'an China; ^2^ Key Laboratory of Insect Developmental and Evolutionary Biology, Institute of Plant Physiology and Ecology, Shanghai Institutes for Biological Sciences Chinese Academy of Sciences Shanghai China

**Keywords:** *Bombyx mori*, CRISPR/Cas9, TCTP

## Abstract

The translationally controlled tumor protein (TCTP) is a highly conserved and multifunctional protein with activities ranging from cytoskeletal regulation to transcription regulation in numerous organisms. In insects, TCTP is essential for cell growth and proliferation. Recently, TCTP has been reported to affect the innate intestinal immune pathway in the *Bombyx mori* silkworm, a lepidopteran model insect. However, the comprehensive physiological roles of TCTP in the silkworm remain poorly understood. Here, we performed functional analysis of *BmTCTP* by using a binary transgenic CRISPR/Cas9 (clustered regularly interspaced short palindromic repeat/RNA‐guided CRISPER‐associated protein 9 nucleases) system. Disruption of *BmTCTP* led to developmental arrestment and subsequent lethality in third instar larvae. Histological analysis revealed that growth impairment originated from decreased cell size, and the proliferation and differentiation of intestinal epithelial cells were also affected. RNA‐seq analysis revealed that genes involved in carbohydrate metabolism, lipid metabolism and digestive system pathways were significantly affected by *BmTCTP* depletion. Together, the results demonstrated that *BmTCTP* plays a key role in controlling larval growth and development.

## Introduction

The translationally controlled tumor protein (TCTP) is highly expressed in tumor cells (Gross *et al*., 1989) and was first identified in mouse erythroleukemia (Yenofsky *et al*., 1983; Bommer & Thiele, 2004). TCTP interacts with many cytoplasmic binding partners and consequently participates in cell growth, proliferation, tumor invasion and apoptosis (Hsu *et al*., 2007; Kloc *et al*., 2012; Amson *et al*., 2013; Acunzo *et al*., 2014). In mice, heterozygous TCTP mutants are viable and fertile, whereas homozygous mutants are embryonically lethal, owing to the role of TCTP in regulating cell proliferation (Chen *et al*., 2007). In *Drosophila*, tissue‐specific decreases in *dTCTP* result in smaller organs via decreases in both cell size and cell number, whereas ubiquitous depletion of *dTCTP* causes lethality during early stages of development, thus revealing the central role of this gene in cell growth and proliferation through regulation of the target of rapamycin signaling pathway (Hsu *et al*., 2007). TCTP also plays important roles in the DNA damage response (DDR) and cell cycle checkpoint to control organ size and chromosome stability in *Drosophila* (Hong & Choi, 2013). Conserved roles of TCTP in growth regulation have also been revealed in plant and mammalian species (Dong *et al*., 2009; Brioudes *et al*., 2010).

The silkworm *Bombyx mori* is one of the most economically important insects and served as a model for lepidopteran insects. High and constitutive *TCTP* transcription have been reported in the silkworm (Lee *et al*., 2004). In the midgut, *BmTCTP* is equally distributed inside the intestinal epithelial cells, but a small quantity has been observed in the peritrophic membrane (PM) (Wang *et al*., 2013a). RNA interference (RNAi) knockdown in the larval midgut results in suppression of intestinal innate immunity after oral microbial infection in intact animals, and the dynamics of extracellular regulated protein kinases (ERK) phosphorylation is disrupted in the midgut in transgenic silkworms (Hu *et al*., 2015). However, the physiological functions in addition to the immunity of *BmTCTP* have not been explored.

To further elucidate the physiological functions of *BmTCTP* beyond the innate immunity pathway, we used a transgenic clustered regularly interspaced short palindromic repeat/CRISPER‐associated protein 9 (CRISPR/Cas9) system (Li *et al*., 2015; Xu *et al*., 2017) to somatically mutate *BmTCTP*. Depletion of *BmTCTP* induced developmental arrest from the larval third instar and eventually resulted in lethality. Histological analysis demonstrated that growth impairment was the cause by the diminution of the cell size in the fat body and also affected the proliferation and differentiation of columnar cells and goblet cells in the midgut. Furthermore, the PM was ruptured. RNA‐seq and subsequent quantitative real‐time polymerase chain reaction (qPCR) analyses suggested dysregulation of carbohydrate metabolism, lipid metabolism and digestive system pathways, thus resulting in silkworm larval development and growth defects.

## Materials and methods

### Insect strains

The silkworm strain Nistari was used in all experiments. Larvae were reared on fresh mulberry leaves at 25 ± 1°C and 70% ± 10% relative humidity and in 12‐h light/12‐h dark cycles.

### RNA isolation and cDNA synthesis

Total RNA was extracted from different tissues with TRIzol reagent (Invitrogen, Carlsbad, CA, USA) according to the manufacturer's instructions. Then, total RNA was treated with RNase‐free DNase I (Ambion, Austin, TX, USA) to remove genomic DNA, according to the manufacturer's protocol. Briefly, 1 μg RNA was used to generate complementary DNA (cDNA) with a RevertAid First‐Strand cDNA Synthesis kit (Fermentas, Vilnius, Lithuania, EU). The reactions were incubated at 37°C for 1 h and terminated by heating at 70°C for 5 min.

### qPCR analysis

qPCR was performed to investigate the relative expression levels of *BmTCTP* (NCBI Reference Sequence: NM_001044107.1) and the messenger RNAs (mRNAs) of other genes (Table 1) in wild type (WT) or transgenic animals. Quantitative mRNA measurements were performed by using SYBR Green Real‐time PCR Master Mix (TOYOBO, Osaka city, Japan). The reactions were performed as follows: initial incubation at 95°C for 1 min followed by 35 cycles of 95°C for 10 s and 55°C for 20 s. The primers used in qPCR to investigate the mRNA expression levels of *BmTCTP* and other genes are provided in Table 1. *Bmrp49* was used as a reference gene that has been validated for qPCR analysis in *Bombyx* (Tan *et al*., 2013). All qPCR experiments were performed with three biological replicates.

**Table 1:** Primers used in this work. TCTP‐sgRNA‐F1, TCTP‐sgRNA‐F2 and sgRNA‐R were used for template amplification to generate sgRNA. Mu‐TCTP‐F and Mu‐TCTP‐R were used for mutagenesis analysis. The remaining primers were used for detection of targeting sites and quantitative real‐time polymerase chain reaction (qPCR)

Primers name	Primer sequence (5′ to 3′)	Primer purpose	Primer identified	NCBI Reference Sequence
TCTP‐sgRNA1‐F:	TATCGTGCTCTACAAGTGGCTGACGAGGG	Preparaion of sgRNA template	RT‐PCR	NM_001044107.1
	CACGGACTGTTTTAGAGCTAGAAATAG			
TCTP‐sgRNA2‐F:	TATCGTGCTCTACAAGTGGAACTTCAGTTC	Preparaion of sgRNA template	RT‐PCR	
	TTTACTGGTTTTAGAGCTAGAAATAG			
sgRNA‐R:	TAGATATCAAGCTGCTAGAAAAAAAAGCACC	Preparaion of sgRNA template	RT‐PCR	
	GACTCGGTGCC			
Mu‐TCTP‐F:	TTTGACTAGACTTATTAATTTC	Detecting mutations	RT‐PCR	NM_001044107.1
Mu‐TCTP‐R:	TTCAGCCTTGTGATGAGAGAAGCC	Detecting mutations	RT‐PCR	
TCTP‐Qpcr‐F:	ACACATTGTACCTCAAAGACTATATG	qPCR	RT‐PCR	NM_001044107.1
TCTP‐Qpcr‐R:	AGGCGCCTTCTCTTCCAATTTTGCTA	qPCR	RT‐PCR	
ChiR1‐F:	CTTGCCCGCTAACGAAGACTTC	qPCR	RT‐PCR	NM_001043396.1
ChiR1‐R:	ACATGTTTCTCCTCACAGGCTC	qRT‐PCR	RT‐PCR	
Su‐Hy‐F:	ATGAGGGCTACCAAGGTGGTGGT	qPCR	RT‐PCR	AB905205.1
Su‐Hy‐R:	ATCACCAATGCCATCACCATCA	qPCR	RT‐PCR	
Ino‐mul‐F:	ATGAGCGATGCGGCTGAACGAC	qPCR	RT‐PCR	XM_012688495.2
Ino‐mul‐R:	TGGGTTTCAGGATTGTTCCGTT	qPCR	RT‐PCR	
Amylase‐F:	TCGCACCACCATGGTACATCTC	qPCR	RT‐PCR	NM_001173153.1
Amylase‐R:	ATTGGTTGATAGCGCTCCCACCAA	qPCR	RT‐PCR	
FE4‐F:	ACATACCTTATGCCTCTGTCACAGAA	qPCR	RT‐PCR	XM_004924556.2
FE4‐R:	GGTAAATTATCTTCATCCGTATCAGG	qPCR	RT‐PCR	
Lipase 3‐F:	CAACTGCCTTCTTTGTGATGACATCAT	qPCR	RT‐PCR	XM_004931417.3
Lipase 3‐R:	AGGTTTGTCCTGCCCAAGTG	qPCR	RT‐PCR	
Tri‐rol‐F:	ATGCGGAGCCCTTTGGCTTTCCTG	qPCR	RT‐PCR	XM_021350267.1
Tri‐rol‐R:	CTTCGTTGAGCAGTTGCATGTTG	qPCR	RT‐PCR	
GDPD6 F:	ATGCGGGGCCGTGTAATAGCTCTATGG	qPCR	RT‐PCR	XM_021349578.1
GDPD6‐R:	GCCCATTGCAATAGCAAGAGCGTAAGC	qPCR	RT‐PCR	
Trypsin‐A‐F:	ATGGTTTCCGTTCTCACCTTAG	qPCR	RT‐PCR	XM_004927083.3
Trypsin‐A‐R:	GTAGTTGGTGAGAACAATACCAGCGCA	qPCR	RT‐PCR	
Sp1F:	GCGGCCGAGGTACCAGGATT	qPCR	RT‐PCR	NM_001043361.1
Sp1R:	CGGAGCGGGTGTTGGTCAGT	qPCR	RT‐PCR	
SP3‐F:	CGAGGTACCAGGATTGTGGGTGGT	qPCR	RT‐PCR	NM_001043438.1
SP3‐R:	AGCAGCGGTCACAGAGCGGG	qPCR	RT‐PCR	
Glucosidase‐F:	ATGGCTTGGTTAACAACTCTCT	qPCR	RT‐PCR	NM_001043608.1
Glucosidase‐R:	CAGGGCGGGTGTGGGTTAAACGA	qPCR	RT‐PCR	

TCTP, translationally controlled tumor protein; sgRNA, single guide RNA; qPCR, quantitative real‐time polymerase chain reaction; RT‐PCR, real‐time polymerase chain reaction; CHiR, chitinase‐related protein; SU‐Hy, sucrose hydrolase; Ino‐mul, inositol polyphosphate multikinase; FE4, esterase FE4‐like; Tri‐rol, triacylglycerol; GDPD6, glycerophosphoryl diester phosphodiesterase 6; SP, serine protease precursor.

### Plasmid construction and germline transformation

Two *piggyBac*‐based transgenic plasmids were used in the current study: CRISPR/Cas9: *pBac[IE1‐EGFP‐IE1‐Cas9]* (*IE1‐Cas9*) and *pBac[IE1‐DsRed‐U6‐BmTCTP sgRNA1‐U6‐BmTCTP sgRNA2]* (*U6‐TCTP sgRNA × 2*). The first plasmid, *IE1‐Cas9*, expresses the *Cas9* nuclease as previously described (Li *et al*., 2015). In the second plasmid, TCTP sgRNAs were expressed under the control of the silkworm *U6* promoter. Two *U6* promoter sequences were amplified by PCR with the enzyme site *Nhe*I and *Sal*I respectively using the silkworm genomic DNA as the template, and sub‐cloned into the initial plasmid to generate *pBac[IE1‐DsRed‐U6‐NheI‐U6‐SalI]*. The two single guide RNAs (sgRNAs) were amplified using the primer TCTP‐sgRNA1‐F/sgRNA‐R, TCTP‐sgRNA2‐F/sgRNA‐R, and inserted into the *Nhe*I and *Sal*I emzyme sites respectively to generate the final plasmid *U6‐TCTP sgRNA × 2*. The target sites were identified by screening the *BmTCTP* open reading frame, by following the 5′‐GG‐N18‐NGG‐3′ rule (Wang *et al*., 2013b). Subsequently, target sequences with high or low GC content, which might affect targeting efficiency, were excluded. The remaining target sequences were subjected to basic local alignment search tool (BLAST) analysis against the silkworm genome to avoid relevant sequences with high identity. The primers used for plasmid construction are listed in Table 1.

The mix of transformation plasmids and helper plasmid in which the final concentration of each vector was 300 ng/μL, was microinjected the into preblastoderm G0 embryos, and then incubated at 25°C in a humidified chamber for 10–12 days until hatching (Tan *et al*., 2013). Hatched larvae were reared into moths and sibling‐mated or backcrossed to WT moths. G1 progeny were scored for the presence of the marker gene during the embryonic stage under a fluorescence microscope (Nikon AZ100, Tokyo, Japan). For the transgenic CRISPR/Cas9 system, *IE1‐Cas9* lines were crossed with the *U6‐TCTP sgRNA* effector line, and the heterozygote progeny (*△TCTP*) were used in subsequent experiments.

### Mutagenesis analysis

Genomic DNA was extracted from somatic mutant larvae for mutagenesis analysis. PCR amplification was performed by using 50 ng genomic DNA as the template to amplify a 1973‐bp region that encompassed the two CRISPR target sites. PCR products were cloned into the pJET‐1.2 vector (Fermentas, Burlington, ON, Canada) and sequenced. The primers used for mutagenesis analysis are listed in Table 1.

### Paraffin embedding and staining

Midguts of the third larval instar day 2 larvae (L3D2) were dissected on ice and fixed in Qurnah's fixative (ethanol : chloroform : acetic acid = 6 : 3 : 1) overnight. The fixed samples were dehydrated in 100% ethanol three times, treated with xylene three times, and embedded in paraffin. Then, 5‐μm thick sections were cut with a Leica RM2235 microtome. For hematoxylin‐eosin staining (HE), sections were dewaxed using xylene and serially rehydrated with 95%, 80% and 70% ethanol, distilled water and phosphate‐buffered saline (PBS), respectively. The fat bodies were stained with Hoechst 33258, and the cell membranes were stained with red fluorescent probe (DiI). All photographs were obtained by fluorescence microscopy (Olympus BX51, Tojyo, Japan).

### Scanning electron microscopy (SEM) analysis

PM of L3D2 larvae were dissected on ice under a microscope, washed in PBS three times and fixed with FAA solution (1 : 1 : 18 ratio of 37%–40% formaldehyde to acetic acid anhydride to 50% ethanol) overnight. The fixed samples were dehydrated with ethyl alcohol dilutions of 50%, 60%, 70%, 80%, 90%, 95% and 100% in a rotary machine. The PM were dried in a critical‐point dryer and then coated with platinum before observation under a SEM (JEOL, Tokyo, Japan).

### RNA‐Seq analysis

Midguts from three silkworm larvae in *△TCTP* and WT silkworm were randomly selected, collected and stored at −80°C for further analyses. cDNA libraries were constructed with an Illumina TruSeqTM RNA Sample Preparation kit (Illumina, San Diego, CA, USA) following the manufacturer's recommendations. The mRNAs were enriched by using oligo (dT) beads (Illumina). After being mixed with the fragmentation buffer, the mRNAs were fragmented into short fragments (approximately 200 bp). The first strand of cDNA was synthesized by using random hexamer primers. Terminal repair with 3′‐adenylation and adapter addition (Illumina) and PCR were performed for 15 cycles with PE 1.0 and PE 2.0 PCR primers. Sequencing was performed using the Illumina HiSeq 2000 system. The raw data obtained from the sequencing were subjected to quality analysis, filtered, and then the HISAT (Kim *et al*., 2015) and Bowtie2 (Langmead *et al*., 2009) were used compared with the reference genome (http://silkworm.genomics.org.cn/). The expression abundance of individual genes was determined as fragments per kb exon per million fragments mapped (FPKM). Possion Distribution Method was used to screening differentially expressed genes (DEGs) with False Discovery Rate (FDR) ≤ 0.001 and the absolute value of Log2Ratio ≥ 1 as the default threshold to judge the significance of gene expression difference. Pathway assignments were determined following the Kyoto Encyclopedia of Genes and Genomes (KEGG). The Blast2GO (Conesa *et al*., 2005) software to obtain Gene Ontology (GO) annotation of DEGs defined by molecular function, cellular component and biological process ontologies.

### Statistical analysis of data

All data were analyzed using GraphPad Prism (Version 5.01) with one‐way analysis of variance and Dunnett post hoc test analysis. All error bars represent the means ± S.E.M. *P* < 0.05 was used as the significance threshold in all cases.

## Results

### Transgene CRISPR induced somatic mutagenesis of BmTCTP

**Figure 1:**
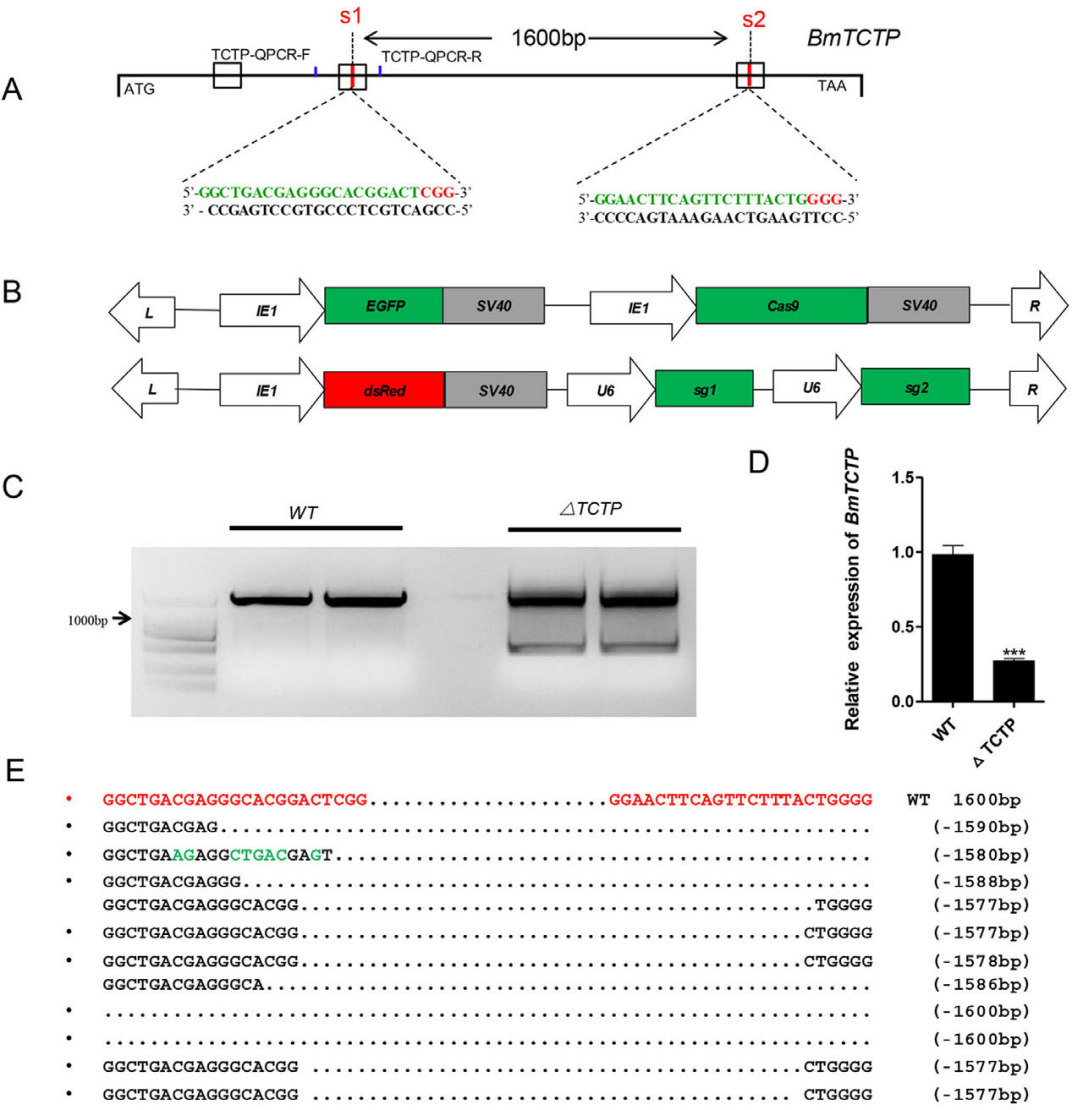
Schematic diagrams of *BmTCTP* transgenic plasmids, *BmTCTP* single guide RNA (sgRNA) and genomic mutagenesis. (A) Schematics of the plasmids *pBac [IE1‐DsRed2‐U6‐BmTCTPsgRNAs]* and *pBac [3xp3‐EGFP‐IE1‐Cas9]*. (B) Schematic diagram of sgRNA‐targeting sites. The boxes indicate the three exons of *BmTCTP*, and the black line represents the gene locus. The sgRNA‐targeting sites S1 and S2 are located on the sense strand of exon‐2 and exon‐3, respectively. The sgRNA‐targeting sequence is indicated in green, and the protospacer adjacent motif (PAM) sequence is indicated in red. (C) Polymerase chain reaction (PCR)‐based amplification of targeted sites in wild type (WT) and *△TCTP* silkworms. (D) Decreased messenger RNA (mRNA) level of *BmTCTP* was detected in *△TCTP* silkworms. The mRNA levels were normalized to those of *Bmrp49*. (E) Various fragment deletions were detected in *△TCTP* silkworms. The red sequence indicates the PAM sequence.

The binary transgenic CRISPR/Cas9 system in *B. mori* has been successfully established to perform somatic loss‐of‐function analysis (Li *et al*., 2015; Xu *et al*., 2017; Zhang *et al*., 2017). To explore the physiological function of the *BmTCTP*, we established two independent transgenic lines expressing Cas9 and *BmTCTP*‐targeting sgRNAs separately (Fig. 1A). This system contained one line (*pBac[IE1‐DsRed2‐U6‐BmTCTPsgRNAs]*) that expressed *BmTCTP* sgRNA under the control of a *B. mori U6* promoter (*U6‐sgRNA*) with the *DsRed* fluorescent marker, and another line *pBac[3xp3‐EGFP‐IE1‐Cas9]* that expressed Cas9 protein under the control of the *B. mori IE1* promoter (*IE1‐Cas9*) with the *EGFP* fluorescent marker. The genomic structure of *BmTCTP* comprises three exons and two introns. In addition, two 23‐bp sgRNA target sites located on exons 2 and 3 were identified and named S1 and S2, respectively. The interspace fragment between the two sites was 1600 bp in length (Fig. 1B).

The U6‐sgRNA plasmid and the helper plasmid were co‐injected into 640 preblastoderm Nistari embryos (G0). Among them, 68% (*n* = 435) of eggs hatched, and 283 larvae survived to the adult stage. All the G0 moths were sibling‐mated, and 12 broods (G1) with *DsRed*‐positive individuals were obtained, as determined through fluorescence microscopy. *U6‐TCTP sgRNA* silkworms (G1) were crossed with *IE1‐Cas9* lines, and 128 somatic mutant animals (G2) were obtained in the *IE1‐Cas9*: *U6‐TCTP sgRNA* (*△TCTP*) progeny.

In *△TCTP* animals, we conducted PCR‐based analysis to investigate the somatic mutations induced by CRISPR. Genomic DNA was extracted from the randomly selected *△TCTP* silkworms and WT animals on day 2 of the third instar (L3D2). The genomic region containing the two targets was PCR amplified by using gene‐specific primers located on either sides of the target (primers shown in Table 1). PCR‐based mutagenesis analysis detected both non‐deletion and deletion mutations (Fig. 1C) and large fragment deletions ranging from 1577–1600 bp (Fig. 1E). Compared with those in WT animals, *BmTCTP* transcripts were also significantly down‐regulated to 30% in *△TCTP* silkworms (Fig. 1D). These results indicated that the transgenic CRISPR/Cas9 system effectively disrupted the *BmTCTP* gene *in vivo*.

### Depletion of BmTCTP induced larval developmental defects

A previous study has demonstrated that silkworms with transgenic RNAi targeting *BmTCTP* exhibit decreased antimicrobial capacity and a disruption of the dynamic ERK phosphorylation in the midgut. To more comprehensively investigate the roles of *BmTCTP* in silkworm growth and development, we compared the developmental time and phenotype of the embryonic period and larval stages between the *△TCTP* silkworms and WT animals. No significant variations were observed in developmental timing and abnormalities in growth during the embryonic period and early larval stages (from first to second larval instar). From the 3rd larval instar, the developmental time of *△TCTP* silkworms was severely delayed and required approximately 8 days, whereas WT animals required only 3 days before entering the 4th larval instar. The body sizes of *△TCTP* silkworms in 3rd instars were significantly smaller than those of the WT animals at the same development time. In total, 99% (*n* = 102) of *△TCTP* larvae died during the 3rd larval instar stage, and the remaining animals died soon after the 4th molting.

**Figure 2:**
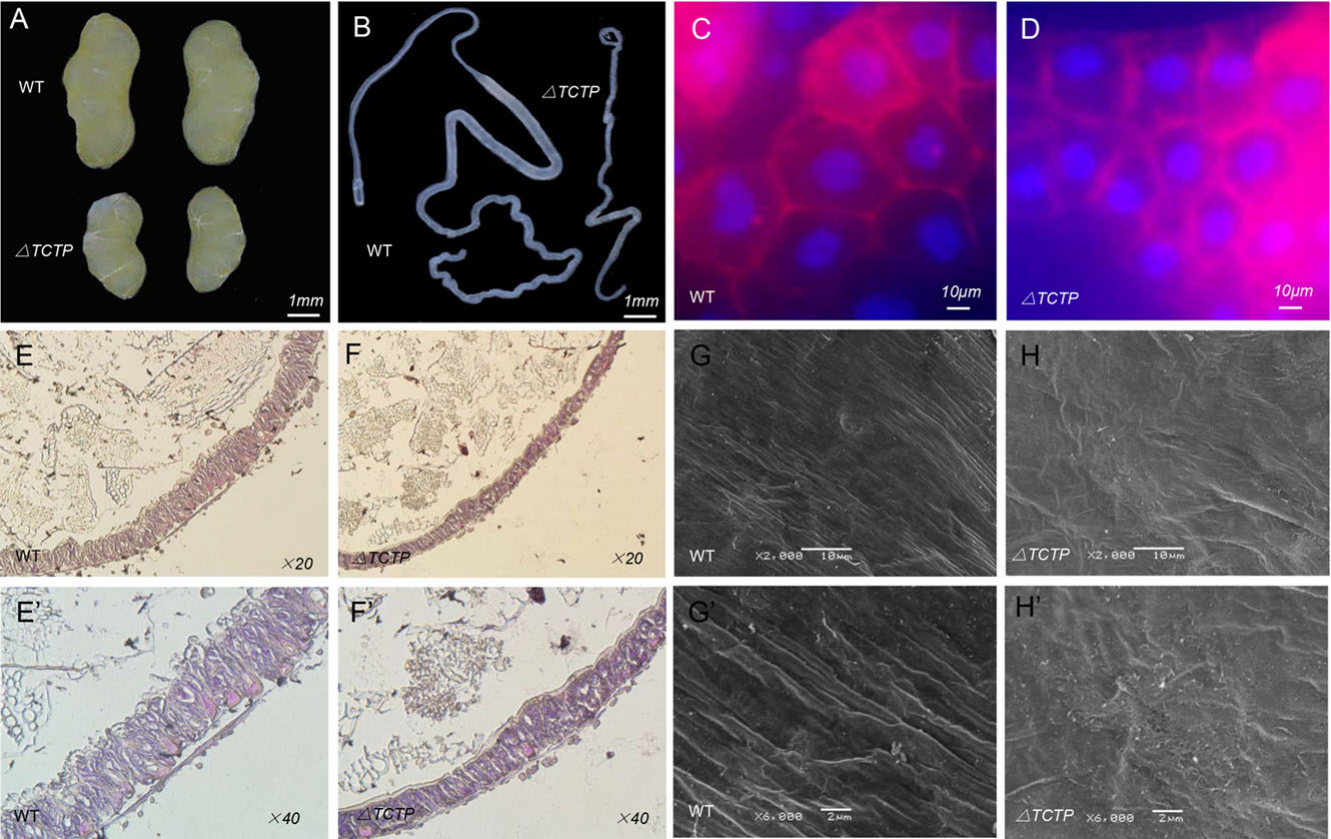
Depletion of *BmTCTP* induced larval growth defects. (A) and (B) *BmTCTP* knock out decreased the gonads and silk gland. (C) and (D) *BmTCTP* knock out caused a significant decrease in cell size. (E, E’ F, F’) Midgut morphology in paraffin sections stained with hematoxylin and eosin (G, G’, H, H’). The peritrophic matrix was abnormal in the mutant silkworm *△TCTP* as examined by scanning electron microscopy (SEM) analysis.

The decreased body size and abnormal development caused by *BmTCTP* knock out may potentially have resulted from abnormal organ development. We next examined phenotypic consequence of fat bodies in L3D2 of *△TCTP* and WT silkworms. As the result, fat bodies in mutant animals showed significantly decreased cell size (Fig. 2C, D). The midgut is another main tissue in the silkworm, and clear abnormal morphologies were observed in *△TCTP* silkworms through histological analysis of the midgut in L3D2. The diameter of the midgut decreased significantly (Fig. 2E, F), and the gut epithelium showed a marked reduction in the number of large, polyploidy nuclei (Fig. 2E’, F’). In addition, the shapes of columnar cells and goblet cells were deformed (Fig. 2E’, F’). We also observed a morphological abnormality of the PM in △*TCTP* silkworms by using SEM and observed numerous dispersive alveoli present on the surface of PM in *△TCTP* silkworms (Fig. 2G, H), whereas the surface of PM was very tight in WT animals (Fig. 2G’, H’). At the same time, the gonads and silk gland also exhibited smaller sizes (Fig. 2A, B). These results demonstrated that *BmTCTP* affects silkworm cell growth and organ development.

### RNA‐Seq and qPCR analysis in △TCTP silkworms

**Figure 3:**
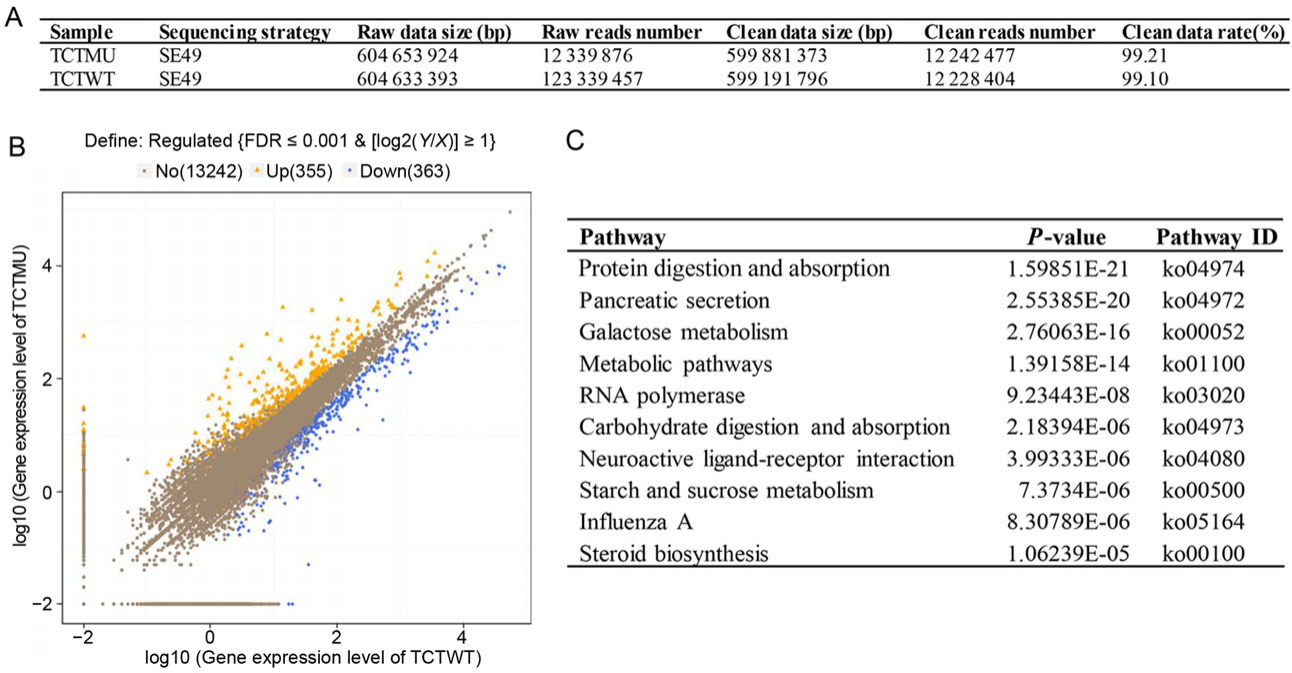
RNA‐Seq analysis. (A) Summary of sequencing data. (B) Statistical chart of significantly differentially expressed genes (DEGs) in *△TCTP* and wild type (WT) silkworms. TCTWT represents WT silkworms, whereas TCTMU represents *△TCTP* silkworms. False discovery rate (FDR) was used to determine the threshold of *P*‐values in multiple tests. We used FDR ≤ 0.001 and the absolute value of log2Ratio ≥ 1 as thresholds to determine significant differences in gene expression. Yellow represents up‐regulated genes, blue represents down‐regulated genes, and gray represents genes without significant differences. (C) The top ten enriched Kyoto Encyclopedia of Genes and Genomes (KEGG) pathways with significant changes were *P*<0.05.

**Figure 4:**
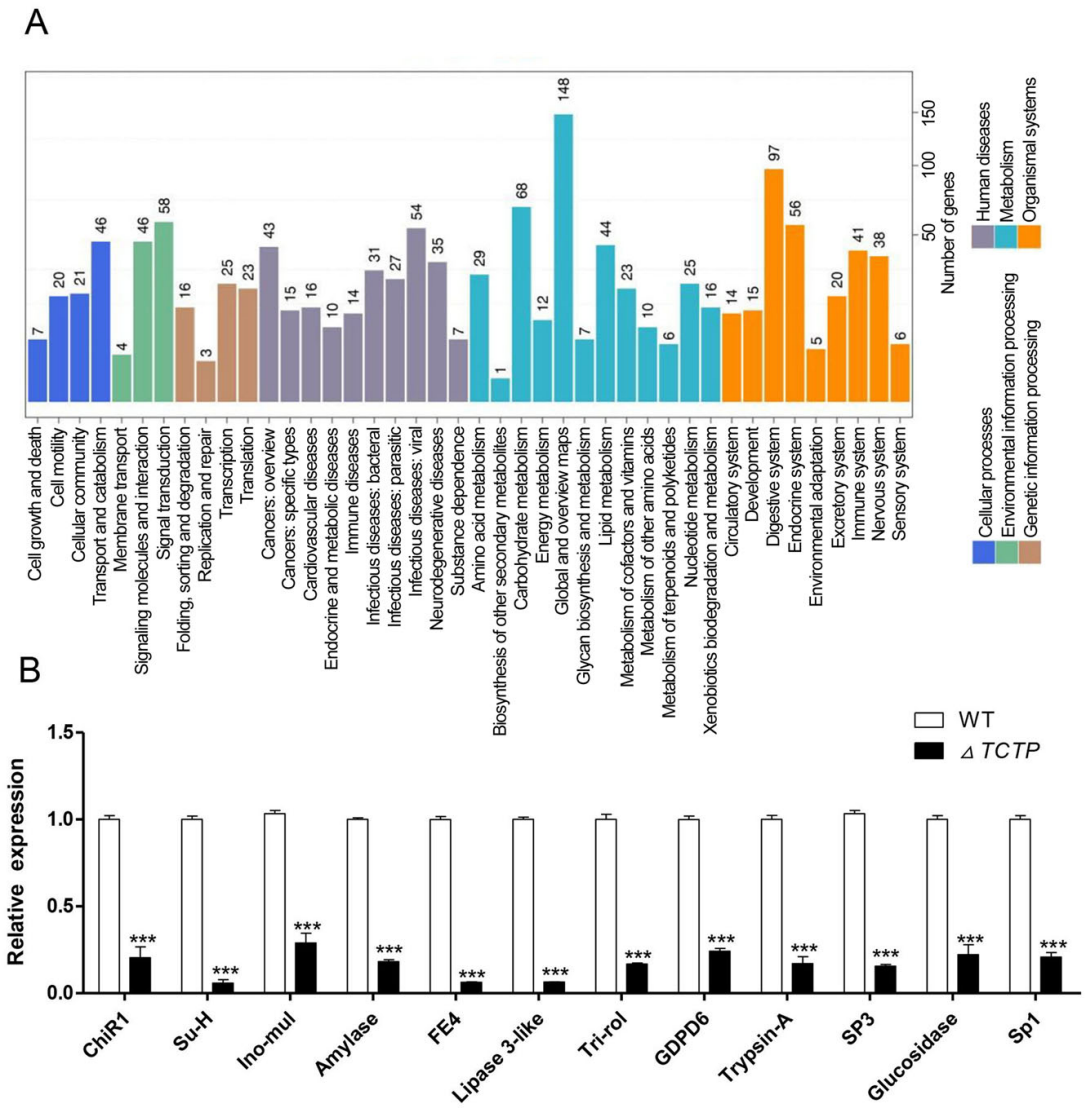
(A) Kyoto Encyclopedia of Genes and Genomes (KEGG) orthology classification of differentially expressed genes (DEGs) in wild type (WT) and *△TCTP* silkworms at the same developmental stage. (B) Quantitative real‐time polymerase chain reaction was used to assess the genes associated with the carbohydrate and protein pathways, lipid metabolism and the digestive system. The mean of the relative expression of the WT was set to 1. Three individuals were analyzed, and the error bars represent the mean ± SE. Asterisks represent statistically significant differences.

To exploit the molecular mechanisms of *BmTCTP* regulation on larval growth and development, the midguts of *△TCTP* silkworms and WT animals were dissected from L3D2 for comparative transcriptional analysis by RNA‐Seq. A summary of sequencing data is shown in Figure 3A. In *△TCTP* silkworms, 13 961 genes were differentially expressed, and 718 genes exhibited significant differences compared with WT animals. Among these genes, 355 were up‐regulated and 363 were down‐regulated, as compared with the ones in WT animals (Fig. 3B). KEGG enrichment analysis revealed that protein digestion and absorption was the most affected pathway in *△TCTP* silkworms (Fig. 3C). In addition, metabolic pathways and organismal systems, such as carbohydrate metabolism, lipid metabolism and the digestive system, were significantly altered between *△TCTP* silkworms and WT animals (Fig. 4A). Further, expression of genes in the immune pathway also showed significant change (Fig 4A). On the basis of the abnormal growth and development, we identified the gene expression patterns related to the metabolism pathway in *△TCTP* silkworms (Fig. 4B). Four genes involved in carbohydrate metabolism, chitinase‐related protein (ChiR), sucrose hydrolase (Su‐H), inositol polyphosphate multikinase (Ino‐mul), and alpha‐amylase (Amylase), were down‐regulated 37‐, 10‐, 5‐ and 4‐fold, respectively, as compared with the expression in WT animals. Four genes involved in lipid metabolism, esterase FE4‐like (FE4), lipase 3‐like (Lip‐3), pancreatic triacylglycerol (Tri‐rol), and glycerophosphoryl diester phosphodiesterase GDPD6 (GDPD6), were down‐regulated approximately 16‐, 15‐, 6‐ and 4‐fold, respectively. The midgut, which is also the main digestive tissue in insects, exhibited an abnormal phenotype in *△TCTP* silkworms. We examined genes related to the digestive system. The genes trypsin alkaline (trypsin‐A) and serine protease precursor 3 (SP3) were both down‐regulated approximately six‐fold. The gene glucosidase precursor (Glucosidase) was down‐regulated approximately eight‐fold, and serine protease precursor 1 (SP1) was down‐regulated four‐fold, as compared with the expression in WT animals. These data suggested that alterations in carbohydrate metabolism, lipid metabolism and the digestive system may result in abnormal growth and development in *△TCTP* silkworms.

## Discussion

*BmTCTP* encodes a highly conserved protein in many organisms and exhibits a ubiquitous expression pattern in the silkworm (Lee *et al*., 2004), thus suggesting its importance in silkworm growth and development. The major contribution of the current study involves the application of a binary transgenic CRISPR/Cas9 system to elucidate the biological functions of *TCTP* in *B. mori*. We demonstrated that depletion of *BmTCTP* severely affects silkworm larval growth and development, thus resulting in larval lethality during the 3rd larval instar.

Somatic mutagenesis of *BmTCTP* induced severe growth and development delay during the 3rd larval stage, thereby resulting in smaller body sizes. Organ size is coordinated with body growth to maintain correct proportions (Mirth & Shingleton, 2012). The sizes of several organs, including the gonads (Fig. 2A), silk gland (Fig. 2B) and midgut (Fig. 2E, E’, F, F’), were significantly smaller than those in WT animals. The fat body is the central metabolic tissue distributed throughout the insect body, and it functions in nutrient synthesis, conversion, utilization and storage (Arrese & Soulages, 2010). In addition, the fat body is also essential for insect growth, development and longevity (Hwangbo *et al*., 2004; Rusten *et al*., 2004). A decreased fat body can have a systemic effect on body growth (Colombani *et al*., 2003, Delanoue *et al*., 2010). We analyzed the fat body by using Hoechst and DiI staining in the 3rd larval instar of *△TCTP* silkworms. Significantly decreased cell size was observed (Fig. 2C, D), revealing that *BmTCTP* functions in cell growth regulation.

A previous report has revealed high *BmTCTP* expression in the silkworm larval midgut and lower expression in PM (Wang *et al*., 2013a). RNAi‐mediated knockdown of midgut *BmTCTP* suppressed the innate intestinal immunity through disruption of dynamic ERK phosphorylation after oral microbial infection. From the RNA‐seq results, 41 genes in the immune pathway showed significant expression differentiation (Fig. 4A), indicating that *BmTCTP* plays an important role in intestinal immune response, as reported before (Hu *et al*., 2015). The midgut is a major player in dietary digestion involving columnar cells and goblet cells. Using histologic analysis, we performed paraffin embedding. Significant decreases in the number of columnar cells and goblet cells were observed (Fig. 2E, E’, F, F’), and the PM exhibited numerous dispersive alveoli (Fig. 2G, G’, H, H’). We also assessed genes related to the digestive system, such as serine proteases, which are the most important digestive enzymes in the lepidopteran larval gut and contribute to approximately 95% of total digestive activity (Srinivasan *et al*., 2006). In our study, trypsin‐A, SP1 and SP3 genes were significantly down‐regulated (Fig. 4B). In the gut, which is also a lipogenic organ, triglyceride packaged together with cholesterol and fat body‐derived carrier proteins form lipoprotein particles, which are trafficked throughout the body (Palm *et al*., 2012). The mRNA expression levels of lipid metabolism pathway‐related genes, such as FE4, Lip‐3, Tri‐rol and GDPD6, in *△TCTP* silkworms were down‐regulated (Fig. 4B). In addition, the mRNA levels of carbohydrate metabolism‐related genes, such as ChiR, Su‐Hy, Ino‐mul and Amylase, were down‐regulated in *△TCTP* silkworms compared with controls (Fig. 4B). These results demonstrated that *BmTCTP* regulates silkworm growth.

Our *in vivo* studies in *B. mori* provided evidence that, similar to *dTCTP* in *Drosophila*, *BmTCTP* is required for maintaining fat body cell size and intestinal cells, thus indicating that TCTP has conserved physiological functions in insects.

## Disclosure

The authors declare no competing financial interests.
